# Defining the frequency of human papillomavirus and polyomavirus infection in urothelial bladder tumours

**DOI:** 10.1038/s41598-018-29438-y

**Published:** 2018-07-26

**Authors:** Matthew A. Llewellyn, Naheema S. Gordon, Ben Abbotts, Nicholas D. James, Maurice P. Zeegers, K. K. Cheng, Andrew Macdonald, Sally Roberts, Joanna L. Parish, Douglas G. Ward, Richard T. Bryan

**Affiliations:** 1 Institute of Cancer and Genomic Sciences, College of Medical and Dental Sciences, University of Birmingham, Birmingham, B15 2TT UK; 2 NUTRIM School for Nutrition and Translational Research in Metabolism, Maastricht University, Maastricht, Netherlands; 3 CAPHRI Care and Public Health Research Institute, Maastricht University, Maastricht, Netherlands; 4 Institute of Applied Health Research, College of Medical and Dental Sciences, University of Birmingham, Birmingham, B15 2TT UK; 5 School of Molecular and Cellular Biology, Faculty of Biological Sciences and Astbury Centre for Structural Molecular Biology, University of Leeds, Leeds, LS2 9JT UK

## Abstract

Given the contradictory nature of the literature regarding the role of human papillomaviruses and polyomaviruses in the pathogenesis of urothelial bladder cancer (UBC), we sought to investigate the frequency of their involvement in a large cohort of primary UBCs. DNA was extracted from 689 fresh-frozen UBC tissues and screened for the presence of high-risk human papillomavirus (HPV) types 16 and 18 and BKV/JCV genomic DNA by qPCR. In positive cases, viral identity was confirmed by Sanger sequencing and viral gene expression was analysed by RT-PCR or immunohistochemistry. All 689 UBCs were negative for HPV18. One UBC from a female patient with areas of squamous differentiation was positive for HPV16. The qPCR data indicated variable levels of polyomavirus in 49 UBCs. In the UBCs with low C_t_s we were able to confirm that 23 were BKV and 6 were JCV by Sanger sequencing. Polyomavirus large T antigen expression was low but detectable in 70% of the sequencing-confirmed polyomavirus positive samples. Thus, in United Kingdom patients, the presence of HPV DNA sequences is extremely rare in UBC (<1% of cases). Polyomavirus DNA (predominantly BKV) is more common in UBC, but still only detectable in 7% of cases and in many of these cases at low copy number. We have performed the largest virus screening to date in UBC, finding that HPV16, HPV18 and HPyV are unlikely to be common causative agents in UBC.

## Introduction

Bladder cancer is estimated to be the 9^th^ most common cancer worldwide^1^. Over 90% of bladder tumours are classed as urothelial bladder carcinomas (UBCs), with 75–80% presenting at non-muscle-invasive (NMIBC) stages and the remainder presenting at muscle-invasive (MIBC) and metastatic stages^2^. Recent sequencing studies have done much to unravel the enormous genomic complexity in UBC^3,4^; however, this explosion of knowledge has not yet led to the effective application of targeted therapies or a complete understanding of what causes the genomic aberrations that initiate and drive UBC. Risk factors which increase the chance of developing bladder cancer include (but are not limited to) age, being male, smoking, occupational exposure to aromatic amines, polycyclic hydrocarbons or heavy metals and ionising radiation^5^. Additionally, chronic inflammation such as that caused by the parasite *Schistosoma haematobium* is a risk factor although usually resulting in squamous cell rather than urothelial carcinoma^5^.

It is estimated that 15% of human cancers are caused by infectious agents^6^. Examples include liver cancer (commonly caused by hepatitis B and C viruses), cervical and head and neck cancers (caused by human papillomaviruses, HPVs), and nasopharyngeal carcinoma and Hodgkin’s and Burkitt’s lymphomas (caused by Epstein-Barr virus, EBV). Although bladder cancer is not widely considered to be a virus-driven cancer, multiple studies have investigated the relationship between oncogenic viruses and UBC: more than 50 studies on HPV and UBC have failed to come to a consensus. Other potentially oncogenic viruses such as EBV and human polyomaviruses (HPyVs) have received less attention. In 2013, Lawrence *et al*.^7^ reported that the mutational signature of UBC is similar to that of two HPV-associated cancers: cervix and head and neck. In 2014 the TCGA detected viral DNA in 6% of the UBCs sequenced^8^. This included three cases of cytomegalovirus, two of HPV16, one of BK polyomavirus (BKV), and one of herpesvirus 6B. Viral transcripts were detected in the BKV positive tumour and one of the HPV16 positive tumours. In these 2 cases, BKV and HPV16 sequences were integrated into *GRB14* and *BCL2L1*, indicating that viruses might play a role in a small percentage of UBCs. We sought to clarify the role of HPVs and HPyVs in the United Kingdom UBC population.

We have used qPCR to screen DNA extracted from the primary UBCs of 689 patients recruited to the West Midlands Bladder Cancer Prognosis Programme (BCPP)^9^ between 2005 and 2011 for the presence of HPV16, HPV18 and HPyVs. This is the largest such screening to date, and the results emphatically support the literature which finds HPV infection to be an extremely rare event in UBC. Our results also support the notion that HPyV can be found in a small proportion of bladder cancers (7%), and find evidence for viral gene expression in a proportion of these. Further investigations are required to determine whether HPyV is a *bona fide* causative factor in some UBCs.

## Methods

### Patient samples and DNA extraction

Patient specimens were collected as part of the Bladder Cancer Prognosis Programme (BCPP)^9^. Immunosuppressed patients were excluded from entering BCPP. All patients gave written informed consent to participate in BCPP, and the work described here was conducted with UK research ethics approval (ref: 06/MRE04/65), and sponsored by the University of Birmingham (BCPP protocol ref: RG_05-088). Hence, all methods were carried out in accordance with the relevant UK guidelines and regulations. Patients were enrolled on the basis of initial cystoscopic findings suggestive of primary UBC. All UBC patients were newly-diagnosed and had not received treatment. Tissues were collected at TURBT and stored at −80 °C. Tissue specimens (25 mg) were macerated and DNA extracted using the DNeasy Blood and Tissue kit (Qiagen). DNA was quantitated using a Qubit (ThermoFisher).

### qPCR

HPV16 and HPV18 were assayed in a multiplex qPCR using primers and probes for HPV16 E6, HPV18 E7 and GAPDH (Sigma). Primer and probe sequences were exactly as described in ref.^10^ and were originally designed to detect only HPV16 or HPV18. All primer and probe sequences are shown in Table S1 and all C_t_ values are shown in Table S2 (Supplemental Information). All qPCRs were carried out in a volume of 20 µl using 10 ng genomic DNA, with primers at 400 nM and probes at 250 nM using TaqMan Universal Master Mix II, no UNG (Applied Biosystems #4440040) using an Applied Biosystems 7500 Fast Real-Time PCR System. The PCR program was 50 °C for 2 min, 95 °C for 10 mins, followed by 45 cycles of 95 °C for 15 s and 55 °C for 1 min. Patient samples were randomly assigned to 11 PCR plates and each plate also contained positive and negative controls, each in triplicate: DNA extracted from UM-SCC-47 cervical cancer cells (HPV16 positive), DNA extracted from HeLa cervical cancer cells (HPV18 positive), DNA extracted from UM-SCC-40 cells (HPV negative) and water blanks. The assay was validated by analysing serial dilutions of HeLa or UM-SCC-47 cell line DNA in UM-SCC-40 cell line DNA (Supplemental Fig. S1). As the assay did not discriminate between HPV16 and HPV18, positive cases were subsequently identified as HPV16 or HPV18 by running separate HPV16 and HPV18 PCRs. PCR products also had their size checked by agarose gel electrophoresis and their identity confirmed by Sanger sequencing.

The qPCR assay for BKV and JCV was adapted from Elfaitouri *et al*.^11^ and used a single degenerate probe and primer pair plus GAPDH primers and probe (Sigma). All other assay parameters were exactly as described above. The assay utilises a conserved region of the VP2 gene and BKV- or JCV-positive tumours with VP2 deletions would not be detected. The assay was validated using pBSSK(II)+ plasmids containing BKV and JCV VP2 sequences (Dundee Cell Products) as shown in Supplemental Fig. S2. To eliminate the risk of sample contamination with the plasmids, the plasmids were only purchased after all samples had been screened. To determine which virus had been detected in bladder tissues, the PCR products were subjected to electrophoresis and the PCR product excised and Sanger sequenced (Eurofins). Primer, probe and plasmid sequences are shown in Table S1. Confirmatory end-point PCRs used 10 ng genomic DNA (0.5 µM primers, sequences as shown in Table S1), Phusion High-Fidelity Mastermix (Thermo Scientific, USA) and gel electrophoresis. The PCR program was 98 °C for 3 min followed by 35 cycles of 98 °C for 15 s, 60 °C for 30 s and 72 °C for 30 s.

### RT-PCR

RNA from frozen specimens and cell lines was extracted using the RNeasy mini kit (Qiagen, Germany) and cDNA synthesised from 0.5 µg RNA with the Superscript Vilo™ kit (Thermo Scientific) according to the manufacturer’s protocol (final volume cDNA = 20 µl). GAPDH PCR was used to verify the success of cDNA synthesis. The presence of viral genes in the cDNA was analysed by PCR using 1 µl cDNA (0.5 µM primers, sequences as shown in Table S1), Phusion High-Fidelity Mastermix (Thermo Scientific, USA) and gel electrophoresis. The PCR program was 98 °C for 3 min followed by 35 cycles of 98 °C for 15 s, 60 °C for 30 s and 72 °C for 30 s.

### Large T antigen immunohistochemistry

De-paraffinisation and antigen retrieval was performed using the citrate buffer/microwave method, before incubating overnight with 2 µg/ml mouse monoclonal antibody against SV40 large T antigen (Abcam, ab16879, cross-reactive with BKV)^12^. Sections were completed using a secondary antibody (ImmPRESS HRP universal detection kit) and visualised using the ImmPACT DAB peroxidase (Vector laboratory, UK). BKV infected/non-infected primary renal proximal tubular epithelial cells were used as controls for antibody specificity (Supplemental Fig. S5). The operator was blinded as to the identity of the samples.

## Results

### HPV

**Figure 1:**
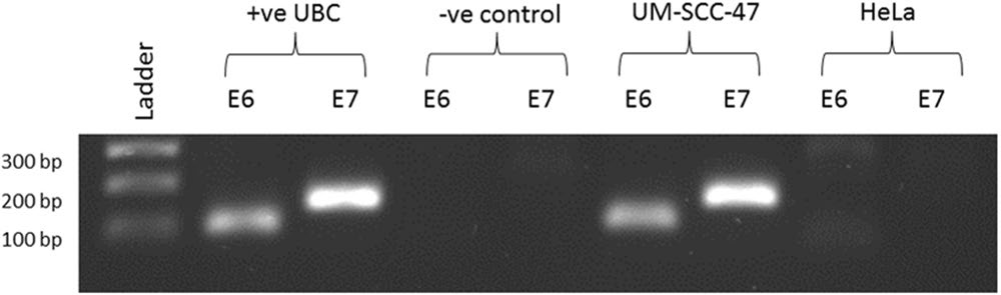
RT-PCR for HPV16 E6 and E7 gene expression. The gel shows PCR products from cDNA synthesised from the HPV positive UBC, a negative DNA control and, for validation purposes, HeLa and UM-SCC-47 cells.

Using the combined assay for HPV16/HPV18 sequences to analyse the DNA extracted from fresh frozen tissue specimens, we obtained a positive result for HPV (C_t_ = 24) in 1 out of the 689 UBCs tested (0.1%) (Table 1) whereas the internal control (GAPDH) was positive in all cases. The HPV positive sample was from a female patient aged 51 and the pathology report noted squamous elements within the tumour. This sample was subsequently assayed for HPV16 and 18 sequences separately and only gave a positive PCR result with HPV16 primers. RT-PCR was used to determine if the HPV16 oncogenes E6 and E7 were expressed in the HPV16 positive UBC: both E6 and E7 mRNAs were detectable (Fig. 1).

**Table 1:** Patient characteristics and viral screening results.

Group	Number	Grade G1/G2/G3	Age	Gender M/F	HPV16 positive	HPV18 positive	BKV positive	JCV positive
*pTis*	4	0/0/4	72	4/0	0	0	0	0
pTa	303	95/158/50	70	234/69	0	0	8	3
pT1	187	3/42/142	72	158/29	0	0	8	3
pT2+	174	0/6/168	72	134/40	1	0	4	0
Stage or grade unknown	21	NA	73	11/10	0	0	3	0

Data shown are the number of individuals in each patient group, grade (1/2/3), median age (yrs), gender (male/female) and the number of individuals positive for HPV16, HPV18 and HPyV.

### Polyomavirus

**Figure 2:**
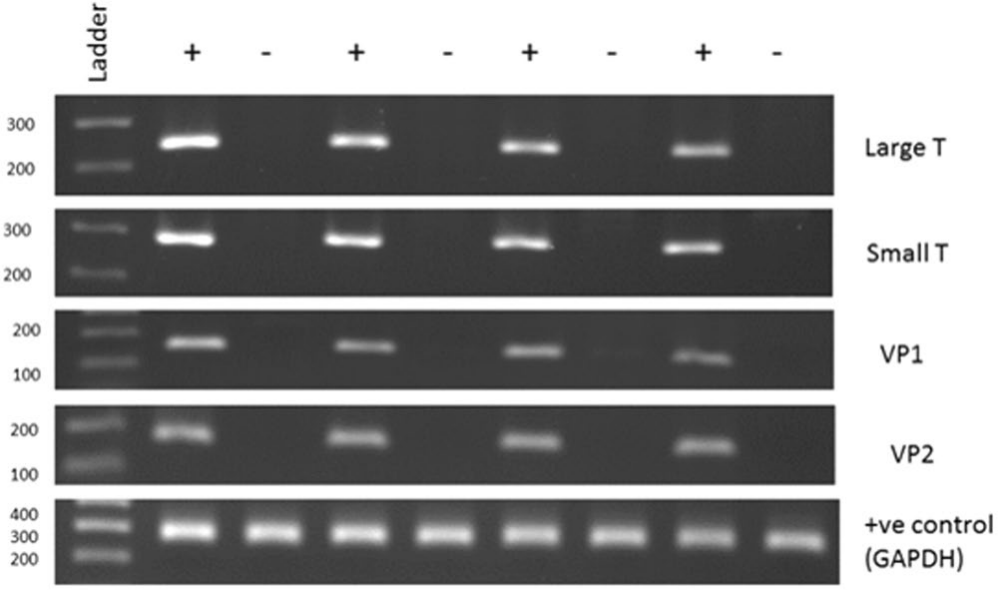
End-point PCR confirmation of polyomavirus qPCR results. The figure shows an agarose gel of PCR products generated using BKV or human GAPDH primers and DNA extracted from tumours. + indicates BKV positive tumours (C_t_s of 17.6, 22.5, 25.3 and 26.2 respectively, left to right) and − indicates BKV negative (C_t_ > 35). The numbers on the left indicate the ladder sizes (bp). The predicted sizes for the 5 amplicons are 250, 281, 161, 164 and 269 bp respectively. The 5 PCRs were run separately (in parallel) and analysed on separate gels.

More than 90% of the samples were negative or below the limit of detection for HPyV. However, HPyV was detected with a C_t_ of <31 in 20 samples, and a further 27 samples showed weak/borderline positivity (C_t_ 31–33). Based on standard curves generated with VP2 gene containing plasmids we estimate that 2 of the most positive UBCs had >100 viral copies per cell, another 3 UBCs had 10–100 copies per cell with the remaining 15 positive cases having 1–10 copies per cell. The 27 weak/borderline cases had a viral copy number <1 copy per cell. All of these samples were subjected to a confirmatory PCR and the products separated by agarose gel electrophoresis. When a sample produced a visible band of the expected size it was excised, the DNA eluted and analysed by Sanger sequencing; in 29 cases a readable HPyV sequence was obtained. BKV was present in 23 samples (mean C_t_ = 28.2) and JCV present in 6 samples (mean C_t_ = 29.9). The presence of multiple regions of the BKV genome was confirmed in a subset of samples using primers specific for BKV VP1, VP2 and Small T antigen (Fig. 2). The age and gender of the HPyV positive cases were similar to the characteristics of the whole patient cohort: median age of 72.5 years old and 26 patients were male and 3 female. Table 1 shows the distribution of the 29 sequencing-confirmed HPyV positive tumours across stages and grades of disease (4 × G1, 9 × G2 and 16 × G3 cases positive). There were no significant differences between the frequency of HPyV detection in different stages or grades of disease (p > 0.05).

**Figure 3:**
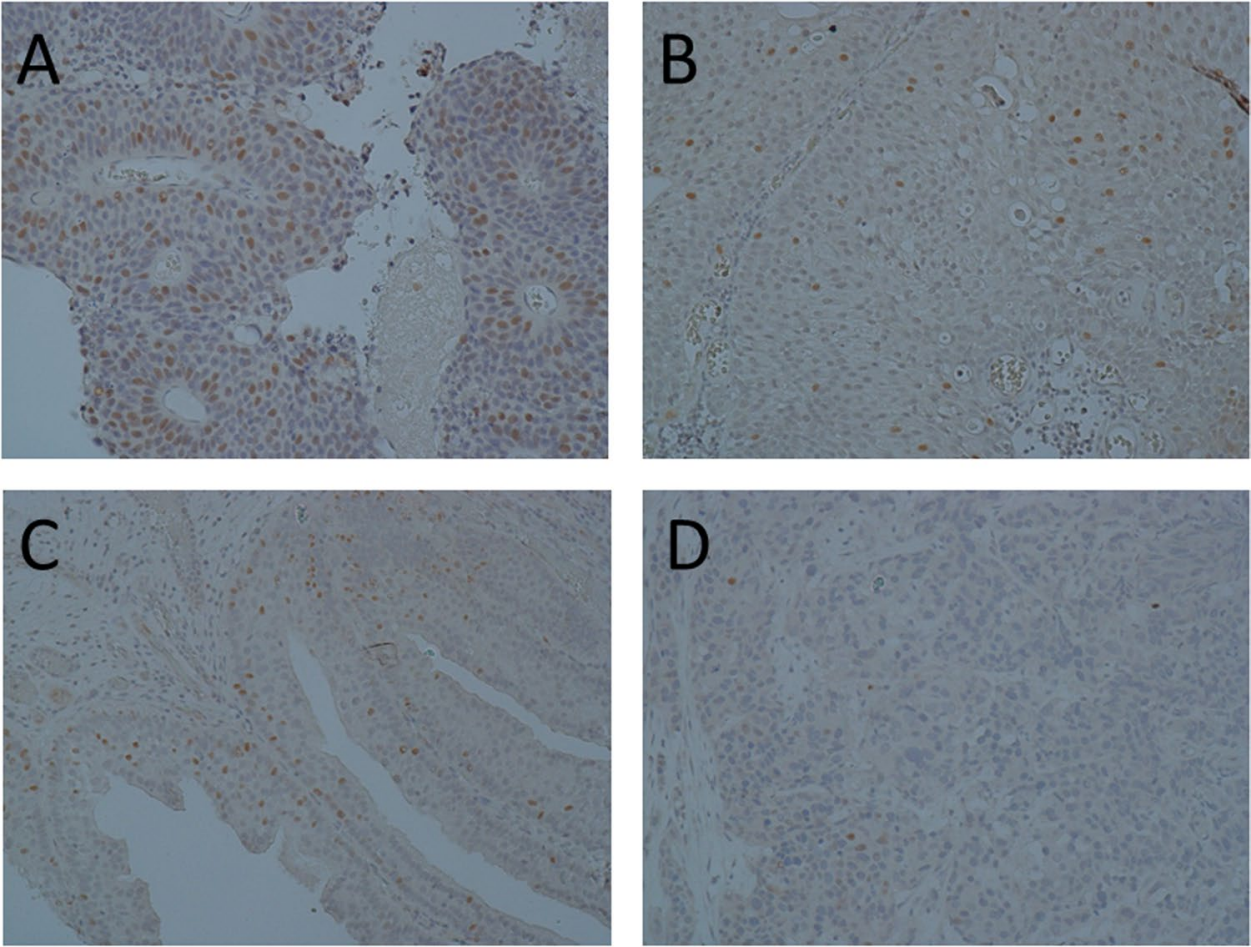
Large T antigen immunohistochemistry. Data are shown for 4 tumours. (**A**) Grade 3 MIBC, C_t_ 17.6, (**B**) grade 2 pTa, C_t_ 21.1, (**C**): grade 2 pTa, C_t_ 29.2, (**D**) grade 3 MIBC, qPCR negative.

Large T antigen expression was analysed by immunohistochemistry in FFPE tissue from 13 of the UBCs which screened positive for HPyV DNA, 8 borderline positive UBCs and 6 negative UBCs. In all cases there was no large T antigen detected in the vast majority of cells. However, nuclear expression was detected in a few scattered cells or patches of cells in 9 of the 13 positive cases, 2 of the 8 borderline cases and 1 of the 6 negative cases. Representative images are shown in Fig. 3. Whilst the correlation between qPCR and IHC data lends support to low-level expression of large T antigen in some of these bladder tumours, we were able to extract reasonable quality RNA (RIN > 7) from several specimens but large T antigen expression was not detectable by qPCR.

## Discussion

We have screened 689 bladder tumours for the presence of HPV16, HPV18, BKV and JCV. We find a complete absence of HPV18 in all tumours, and the presence of HPV16 in only one UBC. Whilst expression of the HPV16 oncogenes was detectable in this sample, the presence of high levels of HPV16 in urothelial carcinomas of the bladder is clearly very rare and as this patient was female we cannot completely rule out that the tumour might have originated in the cervix^13^. Due to limits of analytical sensitivity our data cannot rule out the presence of extremely low levels of HPV16 or HPV18 in bladder tumours, but it is clear that the majority of bladder tumours do not contain HPV16 or HPV18. Thus, for HPV16 or HPV18 to play a significant role in bladder cancer, we have to envisage the scenario that the virus initiates an oncogenic effect which persists after the virus is eliminated. It should, however, be noted that historic infections have been suggested as a cause of APOBEC enzyme activation leading to the APOBEC mutation signature seen in UBCs^7^.

Our data is in line with the lack of detection of HPV DNA in 109 UBCs analysed by PCR in 2015^14^, in 375 UBCs analysed by PCR in 2016^13^, and only three HPV16 and three HPV18 positive cases out of 412 UBCs reported by TCGA^4^. Indeed, our study is the largest single screening of UBCs for HPV16 and 18 to date and strongly supports the hypothesis that these high-risk HPV types are not, or exceedingly rarely, contributors to UBC pathogenesis. Contrarily, so many earlier studies reported moderate to high frequencies of HPV infection in UBC that a recent meta-analysis concluded that 17% of bladder tumours are positive for HPVs, predominantly HPV16 and HPV18^15^. The reasons for this disparity are unclear but may, at least partially, be due to geographical variation with higher HPV incidence in UBC in Asia and Africa compared to Europe and North America^15^.

We detected HPyV DNA at varying levels in approximately 4% of the tissue samples analysed. Thirty of these yielded sufficient PCR product for Sanger sequencing and 23 were identified as BKV and 6 as JCV. Our rate of HPyV positivity is lower than the 14 out of 30 cases (44%) reported by Fioriti *et al*.^16^, but more in line with the 4 out of 30 cases (13%) that Panagiotakis *et al*. reported^17^, and the 4 out of 73 cases (5%) that Rollison reported^18^. Fioriti also reported that, as we have confirmed, BKV is more prevalent than JCV in UBC. Interestingly, Fioriti found no HPyV in bladder tissue from 20 control subjects and Panagiotakis found no HPyV in 30 adjacent normal tissues, the higher frequency in tumours suggesting either that the virus contributes to tumour development, or that the tumour is more susceptible to infection. In our HPyV positive cases, although expression levels were low, immunohistochemistry suggested large T antigen expression in 70% of the HPyV positive UBCs, limited to a few scattered nuclei.

One limitation of our study is that the large T antigen immunohistochemistry data could not be corroborated at the mRNA level. Whilst it is possible that the epitope is expressed but that primer binding sites have been lost during integration, the immunohistochemistry data should be considered preliminary until HPyV gene expression is confirmed by another technique e.g. RNA-Seq. Using genomic methods TCGA detected one case of BKV in 412 UBCs^4,8^. In this UBC the BKV genome was integrated into the human genome and large T antigen mRNA was expressed^4,8,19^. Of the 412 UBCs studied by TCGA, only 136 underwent whole genome sequencing (which should detect BKV DNA), whilst the majority had both exome and RNA sequencing. Exome sequencing library preparation would not be expected to capture viral sequences, and RNA-Seq will only detect viruses if the read depth is sufficient to detect viral mRNA: our data showing that large T antigen expression is limited to a few scattered nuclei suggest that this might not always be the case. Thus, TCGA data may underestimate the frequency of BKV DNA in UBC, but does concur with our data that high levels of BKV gene expression occur extremely rarely in UBC. A second limitation of our study is that there is a continuum of HPyV “positivity” in the qPCR data and it is difficult to differentiate between very low levels and a complete absence of virus. Thus, whilst we report that up to 7% of UBCs may be positive for HPyV, we have only unequivocally demonstrated that 4% of UBCs contain HPyV DNA sequences. It may be worth considering alternative primers or even a probe-based capture and next generation sequencing approach to improve assay sensitivity and specificity in the future.

## Conclusions

We have performed the largest screening to date of HPV16, HPV18 and HPyV in bladder cancer. We found a low prevalence of HPV16 and HPV18 (<1%) and HPyV (7%). Although our results cannot completely exclude a causative role for these viruses in the pathogenesis of UBC, it is clear that high-levels of virus are not persistent driving factors in UBC.

## Electronic supplementary material

Supplemental Information

Table S2
