# Supplementary material for: Defining the frequency of human papillomavirus and polyomavirus infection in urothelial bladder tumours
Source: Sci Rep. 2018 Jul 26;8:11290. doi: 10.1038/s41598-018-29438-y (PMC6062511; doi:10.1038/s41598-018-29438-y)

SREP-17-54867

Defining the frequency of human papillomavirus and polyomavirus infection in urothelial bladder tumours

Matthew A. Llewellyn, Naheema S. Gordon, Ben Abbotts, Nicholas D. James, Maurice P. Zeegers, KK Cheng, Andrew Macdonald, Sally Roberts, Joanna L. Parish, Douglas G. Ward, Richard T. Bryan.

SUPPLEMENTAL INFORMATION

Table S1. Primer, probe and plasmid insert sequences. R=AG; Y=CT; K=GT; S = CG; W=AT; M=AC.

Name	Sequence	Assay
HPV16 E6 Forward	GAACAGCAATACAACAAACC	HPV16 qPCR
HPV16 E6 Reverse	GATCTGCAACAAGACATACA	HPV16 qPCR
HPV16 E6 Probe	FAM-CTGTCAAAAGCCACTGTGTC-BHQ	HPV16 qPCR
HPV18 E7 Forward	GTTGACCTTCTATGTCACGA	HPV18 qPCR
HPV18 E7 Reverse	CAATTCTGGCTTCACACTTA	HPV18 qPCR
HPV18 E7 Probe	FAM-CAATTAAGCGACTCAGAGGAA-BHQ	HPV18 qPCR
GAPDH Forward	GCTCAAGGGAGATAAAATTC	qPCR Control
GAPDH Reverse	CGACCAAATCTAAGAGACAA	qPCR Control
GAPDH Probe	VIC-CCTAGGGCTGCTCACATATT-BHQ	qPCR Control
HPyV Forward	GGGGACCTARTTGCYASTGT	HPyV qPCR
HPyV Reverse	GCAASRGATGCAAKTTSMAC	HPyV qPCR
HPyV Probe	FAM-ACWGGATTTTCAGTRGCTGAAATTGCTGCTGG-BHQ	HPyV qPCR
JCV plasmid insert	GGGGACCTAGTTGCTACTGTTTCTGAGGCTGCTGCTGCCACAGGATTTTCAGTAGCTGAAATTGCTGCTGGAGAGGCTGCTGCTACTATAGAAGTTGAAATTGCATCCCTTGC	HPyV qPCR
BKV plasmid insert	GGGGACCTAGTTGCCAGTGTATCTGAGGCTGCTGCTGCCACAGGATTTTCAGTGGCTGAAATTGCTGCTGGGGAGGCTGCTGCTGCTATAGAAGTTCAAATTGCATCCCTTGC	HPyV qPCR
HPV16 E6 Forward	TGTTTCAGGACCCACAGGAG	RT-PCR
HPV16 E6 Reverse	CTGTTGCTTGCAGTACACACA	RT-PCR
HPV16 E7 Forward	CCGGACAGAGCCCATTACAA	RT-PCR
HPV16 E7 Reverse	TCTGAGAACAGATGGGGCAC	RT-PCR
BKV LT Forward	ATACACAGCAAAGCAGGCAAG	PCR
BKV LT Reverse	GGTGCCAACCTATGGAACAGA	PCR
BKV ST Forward	ACATAGCATGCAAGGGCAGT	PCR
BKV ST Reverse	GAGCTGCCTGGGGAAATCTT	PCR
BKV VP1 Forward	CTGTCACGACAAGCTTCAGT	PCR
BKV VP1 Reverse	ACAGCAGGTAAAGCAGTGGTA	PCR
BKV VP2 Forward	CCTTGCTACTGTAGAGGGCAT	PCR
BKV VP2 Reverse	ACCCTACTTGAGCTAAGGAACT	PCR

Figures S1a and b. HPV qPCR performance. Separate 2-fold dilution series of HeLa DNA (upper panel, HPV18 positive) and UM-SCC-47 DNA (lower panel, HPV16 positive) were made using UM-SCC-40 DNA (HPV negative) as diluent (10 ng total DNA per reaction). Hollow symbols = HPV, filled symbols = GAPDH.

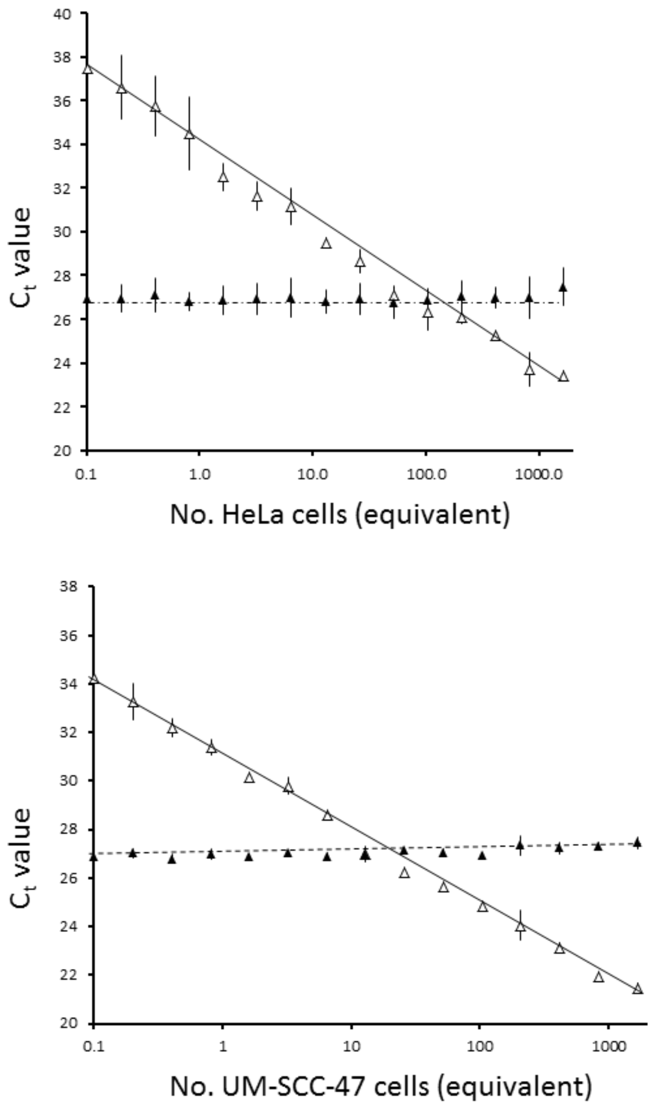

Figure S2. HPyV qPCR performance. Separate 10-fold dilution series of BKV plasmid (filled symbols) and JCV plasmid were made using UM-SCC-40 DNA (HPyV negative) as diluent (10 ng total DNA per reaction).

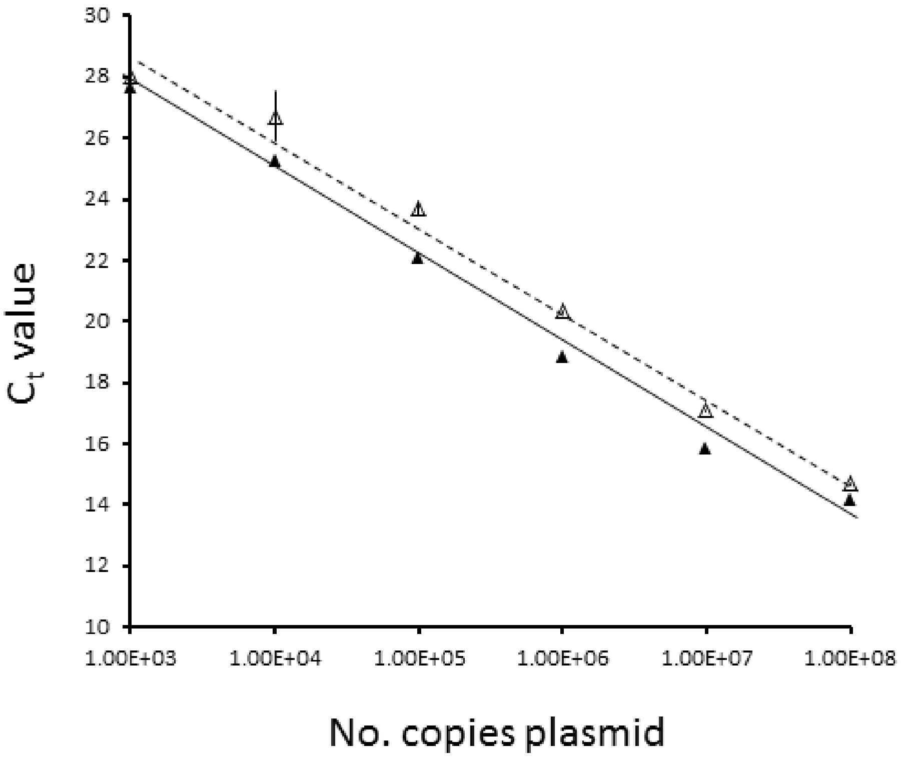

Figure S3. HPyV Sanger sequencing. Representative sequences of HPyV PCR products from DNA extracted from bladder tumours. The arrows indicate the 3 bases which discriminate between BKV and JCV.

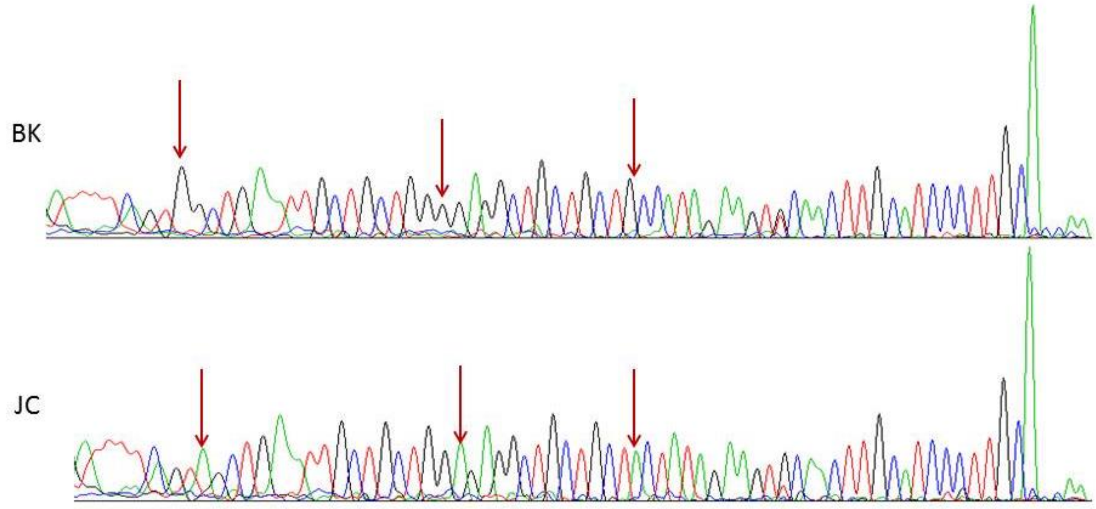

Figure S4. Uncropped versions of gels shown in main manuscript.

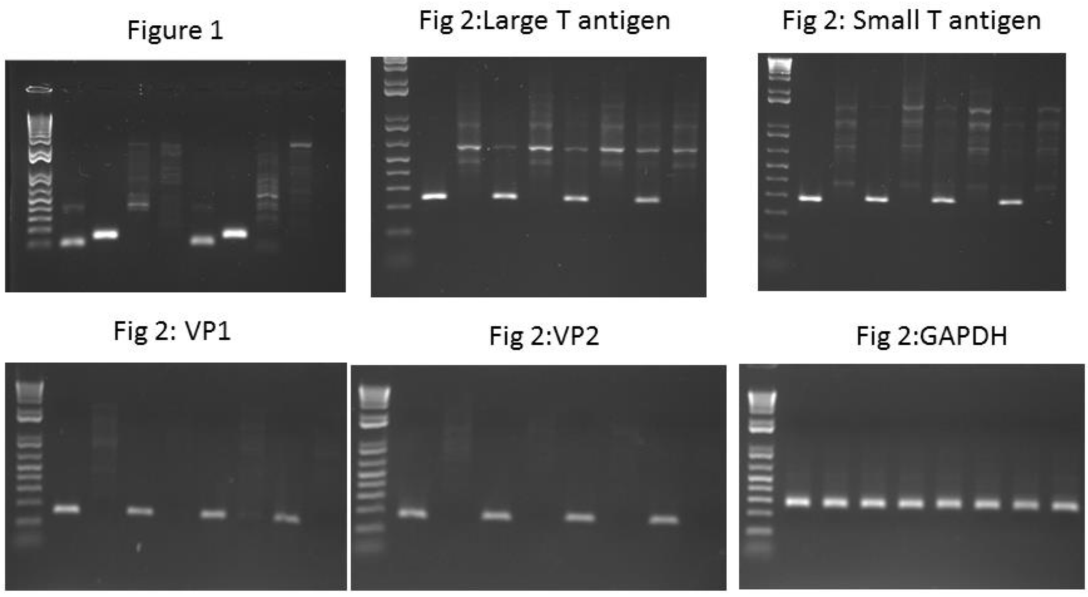

Figure S5. Immunohistochemistry antibody validation. Primary human kidney cells infected (A) or not infected (B) with BKV.

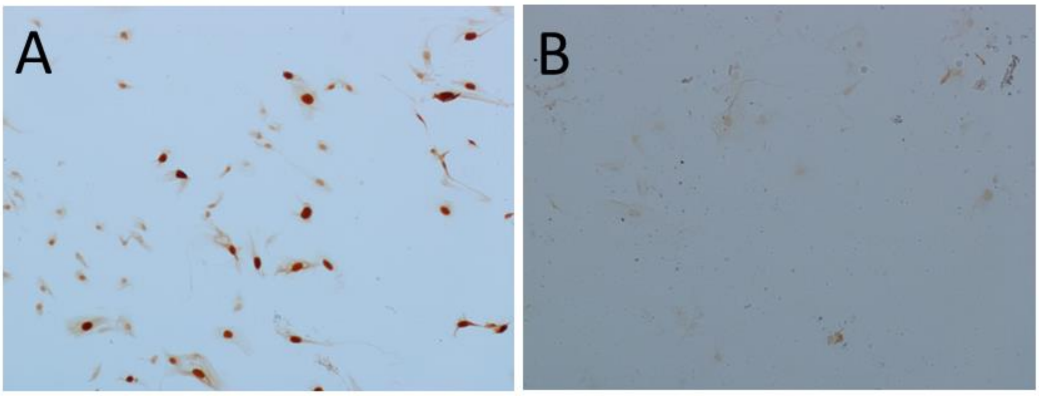

Supplement: Supplementary file 1 — Supplemental Information [file 41598_2018_29438_MOESM1_ESM.pdf]
